# A Study of Hearing Acuity and the Health of the External Auditory Canal Among Earphone Users in Central India

**DOI:** 10.7759/cureus.69664

**Published:** 2024-09-18

**Authors:** Ayushi Ghosh Moulic, Prasad Deshmukh, Shraddha Jain, Sagar S Gaurkar, Jasleen Kakkad, Harshil Dobariya, Jaya Gupta, Amogh Jamadar, Akanksha R Vaidya

**Affiliations:** 1 Otolaryngology - Head and Neck Surgery, Jawaharlal Nehru Medical College, Datta Meghe Institute of HIgher Education and Research, Wardha, IND; 2 Obstetrics and Gynaecology, Kalinga Institute of Medical Sciences, Bhubaneswar, IND

**Keywords:** audiometric testing, background, earphone use, external auditory canal health, hearing loss, noise-canceling earphones, otomycosis

## Abstract

Background and objective

The pervasive use of earphones has raised concerns about its potential impact on hearing acuity and the health of the external auditory canal (EAC). This study aimed to investigate these effects in a sample of individuals in Central India to better understand the associated risks.

Materials and methods

This observational cross-sectional study was conducted in the Department of Otorhinolaryngology, Acharya Vinoba Bhave Rural Hospital, Sawangi Wardha. A total of 130 participants aged 15-35 years were divided into two groups: 65 earphone users and 65 non-earphone users. The data collection involved medical histories, clinical examinations, and audiometric testing, including pure-tone audiometry (PTA), impedance audiometry, and otoacoustic emissions (OAE). Participants completed a questionnaire on earphone usage, and EAC health was assessed via physical examinations and photographic documentation. Statistical analyses included descriptive and inferential statistics, with significance set at p<0.05.

Results

Among earphone users, 45 (69.23%) exhibited hearing loss compared to 11 (16.92%) in non-users (p<0.00001). Noise-canceling earphones were associated with fewer cases of hearing loss (6/45, 13.33%) than non-noise-canceling types (39/45, 86.67%) (p<0.00001). EAC issues were more prevalent in earphone users, with 39 (60%) showing normal conditions versus 52 (80%) in non-users. Impactions of wax, otomycosis, and otitis externa were observed more frequently among earphone users.

Conclusions

The study highlights a significant association between earphone use and increased risk of hearing loss and EAC issues. Noise-canceling earphones may offer some protective benefits. These findings underscore the need for raising awareness and implementing preventive measures to mitigate potential risks associated with prolonged earphone use.

## Introduction

The widespread use of earphones has become common in daily life, largely due to their convenience and the growing availability of portable digital devices. However, concerns have arisen regarding their impact on auditory health. Research indicates that prolonged exposure to high sound levels from earphones can lead to hearing loss, affecting millions globally. Hearing loss due to earphone use is often linked to noise-induced hearing damage, which can result in temporary or permanent auditory impairment [1,2]. Noise-induced hearing loss (NIHL) occurs when exposure to loud sounds damages the delicate hair cells in the cochlea, leading to a gradual decline in hearing ability [3]. The World Health Organization has reported that unsafe listening practices, including using earphones at high volumes, contribute significantly to the rising prevalence of hearing loss in young people [4]. The high-volume levels and extended duration of earphone use are major risk factors for NIHL, with some studies suggesting that even moderate sound levels can be damaging in prolonged exposure [5].

In addition to hearing loss, earphone use may impact the health of the external auditory canal (EAC). Regular use of earphones can lead to conditions such as impacted cerumen, otomycosis, and otitis externa [6]. The constant insertion and removal of earphones can create a favorable environment for microbial growth and earwax accumulation, potentially leading to infections and inflammation [7]. Given these concerns, studies need to evaluate the effects of earphone use on hearing acuity and the health of the EAC. This research aims to provide a comprehensive assessment of these effects among earphone users in Central India, contributing to a better understanding of the risks associated with earphone use and informing preventive strategies.

## Materials and methods

Study setting

The study was conducted at the Department of Otorhinolaryngology, Acharya Vinoba Bhave Rural Hospital, Sawangi Wardha. This setting provided a controlled environment for evaluating the impact of earphone use on hearing and EAC health, allowing for standardized procedures and data collection.

Study design and participants

This observational cross-sectional study aimed to assess the effects of earphone use on hearing and EAC conditions. It involved 130 participants aged 15-35 years, divided into 65 earphone users and 65 non-earphone users. The study used clinical and audiometric assessments to gather comprehensive data to compare the prevalence of hearing impairment and EAC issues between these two groups. The duration of the study was between December 2022 and June 2024. Participants were recruited through a systematic selection process at the Department of Otorhinolaryngology, Acharya Vinoba Bhave Rural Hospital, Sawangi Wardha, following approval from the institutional ethics committee. Eligible participants were between 15 and 35 years old and were categorized based on their earphone usage habits.

Earphone users were defined as individuals who regularly used earphones for at least one hour per day over the past six months, while non-users either did not use earphones at all or used them less than once per month. To address potential confounding factors, participants were asked detailed questions regarding their exposure to other noise sources, such as occupational or environmental noise, to ensure that earphone use was the primary factor under investigation. Only participants with no significant exposure to occupational or environmental noise were included in the final analysis to minimize external noise-related variables. The purpose of earphone usage was also recorded, with participants specifying whether they used earphones for entertainment, professional activities, or other purposes. Additionally, the type of earphones (e.g., in-ear, over-ear, noise-canceling) and the brand or model were documented.

Participants used their earphones, and the study did not standardize the devices. However, the study controlled for variations by collecting detailed information on earphone types, usage patterns, and noise-canceling features. To address the variability in volume levels, participants were asked to self-report the average volume at which they listened to audio on a scale of 1 to 10, with 1 representing very low volume and 10 representing full volume. While subjective, this scale provided an estimate of individual listening habits. Additionally, audiometric testing was used to assess the overall hearing levels and hearing loss, providing objective data on the effects of different volume levels on participants’ auditory health. Exclusion criteria for both groups included individuals with pre-existing ear conditions (e.g., chronic otitis media), a history of ear surgery, congenital hearing impairment, or significant noise exposure from other sources.

Data collection

The data collection process was meticulously organized to ensure a comprehensive and accurate assessment of the impact of earphone use on hearing acuity and EAC health. Recruitment began after obtaining approval from the institutional ethics committee. Participants were enrolled based on strict inclusion and exclusion criteria and informed written consent was collected from all participants to ensure they were fully aware of the study's objectives and procedures, maintaining ethical standards and participant autonomy. Following enrollment, a comprehensive baseline assessment was performed for each participant. This included gathering detailed medical histories and conducting thorough ear, nose, and throat examinations to establish a reference point for the participant’s hearing and ear health. This baseline was critical to evaluate potential changes attributed to earphone use during the study.

Participants were required to complete a detailed questionnaire that collected essential information on earphone usage habits, including the duration of use (measured in hours per day), volume settings (self-reported on a scale of 1 to 10), and the primary purpose of earphone use (e.g., entertainment, professional activities, phone calls). Additionally, participants were asked if their earphones had noise-canceling features, as this could potentially reduce the volume levels required for clear listening in noisy environments. To control for the influence of this factor, the presence or absence of noise-canceling technology was recorded, and the statistical analysis later accounted for this difference to prevent confounding in the results. Although participants used their personal earphones, detailed information on the type and model of earphones was collected to ensure that differences in earphone technology (e.g., noise-canceling) were properly considered during the analysis.

The same clinical and audiometric assessments were applied to all participants, regardless of earphone use status, to minimize bias and ensure a standardized evaluation. Audiometric testing included pure-tone audiometry (PTA), conducted using the ALPS Advanced Digital Audiometer AD2100, to measure hearing thresholds across various frequencies, and impedance audiometry, performed with a tympanometer, to assess middle ear function [8]. Additionally, otoacoustic emissions (OAE) testing was conducted using a handheld OAE screener to evaluate the cochlear function and hair cell integrity, both of which could be affected by prolonged earphone use [9]. The potential impact of EAC conditions, such as otomycosis, impacted wax, or other abnormalities, was evaluated through regular physical examinations. Photographic documentation of the EAC was performed in certain cases to visually record and assess changes over time. These examinations were critical in identifying any conditions that could be linked to earphone use.

To ensure data integrity and reliability, all collected information, including clinical findings, audiometric results, and questionnaire responses, was entered into a standardized and confidential database. This structured approach facilitated accurate analysis and maintained data confidentiality. Additionally, all audiometric equipment was regularly calibrated to minimize measurement bias, particularly in PTA readings. Participants were also monitored for any signs of malingering to ensure the reliability of the test results. While noise-canceling earphones were identified as a variable, the analysis accounted for this difference by controlling for its effect on volume levels. However, it is acknowledged that variations in earphone types and technologies could introduce differences between groups. Therefore, these factors were carefully considered during data analysis to ensure the robustness and credibility of the study’s findings.

Statistical analysis

The statistical analysis for the study involved several key steps to assess the impact of earphone use on hearing and ear health. Data cleaning and preparation were performed to ensure accuracy and consistency, including handling outliers and missing values. Descriptive statistics summarized key features of the dataset, such as means and percentages, providing an overview of trends and patterns. Inferential statistics, including Chi-square tests for categorical variables and t-tests for continuous variables, were used to identify significant differences between earphone users and non-users. The significance level was set at 5% (p<0.05), with results below this threshold considered statistically significant. R studio version 4.3.1 facilitated complex analyses, ensuring accurate and reliable results.

Ethical considerations

The study adhered to ethical standards by obtaining approval from the institutional ethics committee and ensuring informed consent from all participants. The data were anonymized to maintain confidentiality, and participants were assured of their right to withdraw without consequence. Non-invasive procedures were used to minimize discomfort, and equipment was regularly calibrated to ensure accurate results. These measures protected participants' rights and welfare throughout the study. The protocol has been published in F1000Research [10].

## Results

As shown in Table 1, the cohort had more female participants (64.61%) than males (35.38%), indicating a gender imbalance that may affect the overall results and interpretations.

**Table 1:** Gender distribution of the participants

Sex	Frequency (%)
Female	84 (64.61%)
Male	46 (35.38%)

As presented in Table 2, the highest proportion of earphone users was in the 15-20 age group (55.38%), with usage declining in older age groups. This suggests that earphone use is more prevalent among younger individuals. The data in Table 3 highlights widespread personal listening device use, with earphones and higher volume levels being common, and a notable minority reporting ear discomfort or hearing issues.

**Table 2:** Participants and PLD use PLD: personal listening devices

Age group, years	Users, n (%)	Non-users, n (%)	Total, n (%)
15-20	36 (55.38%)	44 (67.69%)	80 (61.53%)
20-25	23 (35.38%)	14 (21.53%)	37 (28.46%)
25-30	4 (6.15%)	3 (4.61%)	7 (5.38%)
30-35	2 (3.08%)	4 (6.15%)	6 (4.61%)
Total	65 (100%)	65 (100%)	130 (100%)

**Table 3:** Responses to the questionnaire on personal listening device usage

Question	Response options	Responses, n (%)
Do you use personal listening devices regularly?	Yes	65 (100%)
	No	0 (0%)
What type of personal listening devices do you use?	Headphones	25 (38.46%)
	Earphones	40 (61.53%)
What do you use your personal listening device for?	Music	40 (61.53%)
	Phone calls	10 (15.38%)
	Both	15 (23.07%)
For how long do you use your personal listening device on average in a day?	1-2 hours	10 (15.38%)
	2-4 hours	25 (38.46%)
	4-6 hours	15 (23.07%)
	More than 6 hours	15 (23.07%)
On an average scale of 1 to 10, what is the normal range of volume that you listen to?	1-3	5 (7.69%)
	3-7	25 (38.46%)
	7-10	30 (46.15%)
	Full volume always	5 (7.69%)
Do you share your personal listening device with others?	Yes	5 (7.69%)
	No	40 (61.53%)
	Sometimes	20 (30.76%)
Do you feel a ringing sensation in your ears after removing the personal listening device?	Yes	5 (7.69%)
	No	45 (69.23%)
	Sometimes	15 (23.07%)
Does your ear hurt on removing the personal listening device after use?	Yes	10 (15.38%)
	No	55 (84.61%)
Does your ear itch regularly after using your personal listening device?	Yes	5 (7.69%)
	No	60 (92.30%)
Do you feel that recently you have difficulty hearing or are experiencing hearing loss?	Yes	10 (15.38%)
	No	55 (84.61%)
Do you feel that the amount of ear wax has increased in your ears recently?	Yes	5 (7.69%)
	No	60 (92.30%)
Do you have a noise-canceling feature in your earphones?	Yes	20 (30.76%)
	No	45 (69.23%)

As illustrated in Table 4, students represented the majority of earphone users (50.7%), followed by software engineers (13.8%). This indicates that using earphones is common among students and professionals in sedentary work environments.

**Table 4:** Occupation-wise distribution of participants

Occupation	Users, n (%)	Non-users, n (%)
Engineer (non-software)	6 (9.2%)	6 (9.2%)
Software engineer	9 (13.8%)	9 (13.8%)
Student	33 (50.7%)	25 (38.4%)
Nurse	6 (9.2%)	9 (13.8%)
Homemaker	6 (9.2%)	11 (16.9%)
Teacher	5 (7.9%)	5 (7.9%)
Total	65 (100%)	65 (100%)

As depicted in Table 5, earphone users had a significantly higher prevalence of hearing loss (69.23%) than non-users (16.92%), suggesting a strong association between earphone use and hearing impairment.

**Table 5:** Hearing status in PLD users vs. non-users PLD: personal listening devices

Hearing status	Users, n (%)	Non-users, n (%)	Chi-square	P-value
Normal Hearing	20 (30.76%)	54 (83.07%)	36.2645	<0.00001
Hearing Loss	45 (69.23%)	11 (16.92%)		

Participants with noise-canceling earphones had a lower incidence of hearing loss (13.33%) than those without this feature (86.67%) (Table 6). This indicates that noise-canceling technology may mitigate some of the negative effects of earphone use on hearing.

**Table 6:** Noise-canceling feature in PLD and hearing status PLD: personal listening devices

Noise-canceling feature	Normal, n (%)	Hearing loss, n (%)	Chi-square	P-value
Present	16 (80%)	6 (13.33%)	27.4841	<0.00001
Absent	4 (20%)	39 (86.67%)		
Total	20 (100%)	45 (100%)		

As shown in Table 7, earphone users had a higher prevalence of impacted wax (18.46%), otomycosis (9.23%), and otitis externa (12.30%) compared to non-users. This suggests that earphone use is associated with a higher incidence of external auditory canal issues.

**Table 7:** External auditory canal health in users vs. non-users

User status	Users, n (%)	Non-users, n (%)
Normal	39 (60%)	52 (80%)
Impacted Wax	12 (18.46%)	9 (13.84%)
Otomycosis	6 (9.23%)	2 (3.07%)
Otitis Externa	8 (12.30%)	2 (3.07%)
Total	65 (100%)	65 (100%)

## Discussion

The study's findings offer critical insights into the impact of earphone use on hearing acuity and the health of the EAC. We observed a notably higher prevalence of hearing loss among earphone users than non-users (69.23% vs. 16.92%, p<0.00001). This significant disparity underscores the risks associated with prolonged exposure to high volumes through earphones, corroborating existing literature highlighting the dangers of sustained loud sound exposure [11,12]. Furthermore, the heightened risk of hearing impairment is particularly evident in users whose earphones lack noise-canceling features. The absence of these features correlates with a higher incidence of hearing loss, suggesting that the inability to block out external noise may lead users to increase the volume to harmful levels (p<0.00001). These findings emphasize the importance of noise-canceling technology in reducing the risk of hearing damage by allowing users to listen at lower volumes while still achieving clear audio.

In addition to hearing loss, the study sheds light on the effects of earphone use on EAC health. Earphone users showed a higher frequency of impacted wax (18.46% vs. 13.84%), otomycosis (9.23% vs. 3.07%), and otitis externa (12.30% vs. 3.07%) compared to non-users. These observations align with previous studies that suggest earphone use can contribute to cerumen accumulation and increase the likelihood of external ear infections [13,14]. The physical presence of earphones in the ear canal creates an environment that may trap moisture and heat, promoting conditions conducive to fungal growth, thereby heightening the risk of otomycosis [7]. This aspect of earphone use, particularly in humid environments or among individuals prone to ear canal infections, necessitates further attention to hygiene practices and modifying the design of earphones to minimize these risks.

The study also provides demographic insights, indicating that earphone use is more prevalent among younger individuals, especially students. This trend supports previous research that younger populations are more inclined to use earphones at high volumes, potentially due to lifestyle and recreational habits [15]. Additionally, the higher incidence of earphone use among individuals in engineering and software-related professions may reflect occupational demands, where extended earphone use is common during work or study [16]. These findings highlight the need for targeted interventions and educational campaigns focusing on younger populations and professionals at higher risk of hearing damage due to prolonged and frequent earphone use.

Lastly, the study underscores the importance of noise-canceling features as a preventive measure against hearing loss. The significantly lower prevalence of hearing impairment among users of noise-canceling earphones suggests that these features can effectively reduce the need for high volume levels by blocking out ambient noise, thus minimizing the overall exposure to potentially harmful sound levels [17]. This evidence advocates for adopting noise-canceling technology as a standard feature in earphones to protect users' hearing health, particularly in noisy environments.

Limitations

The study's cross-sectional design limited its ability to determine causal relationships and assess the long-term effects of earphone use. Self-reported data may have introduced recall bias, and the findings may not be generalizable beyond the specific age group and region studied. Additionally, factors like earphone type and environmental noise were not fully controlled, which could impact the results [18]. Future research should involve longitudinal studies to better understand the long-term effects of earphone use on hearing and ear health. Additionally, investigating the impact of different earphone designs and materials on auditory health could provide further insights into mitigating the risks associated with earphone use.

## Conclusions

This study demonstrates a significant correlation between earphone use and adverse effects on hearing acuity and the health of EAC. Earphone users exhibited a notably higher prevalence of hearing loss than non-users, with those using non-noise-canceling earphones experiencing more severe outcomes. Additionally, earphone users were more likely to have EAC issues, such as impacted wax and otomycosis. These findings suggest that prolonged and frequent earphone use can compromise ear health and auditory function. The potential protective effect of noise-canceling features highlights the importance of adopting earphones with such technology. Overall, the results advocate for increased public awareness and preventive strategies to reduce the risks associated with earphone use, emphasizing the need for safe listening practices to preserve hearing health.

## Appendices

Questionnaire

Do you use personal listening devices regularly? A) Yes B) No

What type of personal listening devices do you use? A) Headphones B) Earphones

What do you use your personal listening device for? A) Music B) Phone calls C) Both

For how long do you use your personal listening device on average in a day? A) 1-2 hours B) 2-4 hours C) 4-6 hours D) More than 6 hours

On an average scale of 1 to 10 (10 being the highest), what is the normal range of volume that you listen to while using your personal listening device? A) 1-3 B) 3-7 C) 7-10 D) Full volume always

Do you share your personal listening device with others? A) Yes B) No C) Sometimes

Do you feel a ringing sensation in your ears after removing the personal listening device? A) Yes B) No C) Sometimes

Does your ear hurt on removing the personal listening device after use? A) Yes B) No

Does your ear itch regularly after using your personal listening device? A) Yes B) No

Do you feel that recently you have difficulty hearing or are experiencing hearing loss? A) Yes B) No

Do you feel that the amount of ear wax has increased in your ears recently? A) Yes B) No

Do you have a noise-canceling feature in your earphones? A) Yes B) No
